# Regulation of β-cell death by ADP-ribosylhydrolase ARH3 via lipid signaling in insulitis

**DOI:** 10.1186/s12964-023-01437-1

**Published:** 2024-02-21

**Authors:** Soumyadeep Sarkar, Cailin Deiter, Jennifer E. Kyle, Michelle A. Guney, Dylan Sarbaugh, Ruichuan Yin, Xiangtang Li, Yi Cui, Mireia Ramos-Rodriguez, Carrie D. Nicora, Farooq Syed, Jonas Juan-Mateu, Charanya Muralidharan, Lorenzo Pasquali, Carmella Evans-Molina, Decio L. Eizirik, Bobbie-Jo M. Webb-Robertson, Kristin Burnum-Johnson, Galya Orr, Julia Laskin, Thomas O. Metz, Raghavendra G. Mirmira, Lori Sussel, Charles Ansong, Ernesto S. Nakayasu

**Affiliations:** 1 Biological Sciences Division, Pacific Northwest National Laboratory, Richland, WA 99354 USA; 2 Barbara Davis Center for Diabetes, University of Colorado Anschutz Medical Center, Aurora, CO 80045 USA; 3 Department of Chemistry, Purdue University, West Lafayette, IN 47907-2084 USA; 4 Environmental and Molecular Sciences Laboratory, Pacific Northwest National Laboratory, Richland, WA 99354 USA; 5 NanoString Technologies, Seattle, WA 98109 USA; 6 Endocrine Regulatory Genomics, Department of Experimental & Health Sciences, University Pompeu Fabra, 08003 Barcelona, Spain; 7 Center for Diabetes and Metabolic Diseases and the Herman B Wells Center for Pediatric Research, Indiana University School of Medicine, Indianapolis, IN 46202 USA; 8 ULB Center for Diabetes Research, Université Libre de Bruxelles (ULB), 1070 Brussels, Belgium; 9 Centre for Genomic Regulation (CRG), The Barcelona Institute of Science and Technology, 08003 Barcelona, Spain; 10 Kovler Diabetes Center and Department of Medicine, The University of Chicago, Chicago, IL 60637 USA; 11 Department of Biostatistics and Informatics, University of Colorado Anschutz Medical Center, Aurora, CO 80045 USA

## Abstract

**Background:**

Lipids are regulators of insulitis and β-cell death in type 1 diabetes development, but the underlying mechanisms are poorly understood. Here, we investigated how the islet lipid composition and downstream signaling regulate β-cell death.

**Methods:**

We performed lipidomics using three models of insulitis: human islets and EndoC-βH1 β cells treated with the pro-inflammatory cytokines interlukine-1β and interferon-γ, and islets from pre-diabetic non-obese mice. We also performed mass spectrometry and fluorescence imaging to determine the localization of lipids and enzyme in islets. RNAi, apoptotic assay, and qPCR were performed to determine the role of a specific factor in lipid-mediated cytokine signaling.

**Results:**

Across all three models, lipidomic analyses showed a consistent increase of lysophosphatidylcholine species and phosphatidylcholines with polyunsaturated fatty acids and a reduction of triacylglycerol species. Imaging assays showed that phosphatidylcholines with polyunsaturated fatty acids and their hydrolyzing enzyme phospholipase PLA2G6 are enriched in islets. In downstream signaling, omega-3 fatty acids reduce cytokine-induced β-cell death by improving the expression of ADP-ribosylhydrolase ARH3. The mechanism involves omega-3 fatty acid-mediated reduction of the histone methylation polycomb complex PRC2 component Suz12, upregulating the expression of *Arh3*, which in turn decreases cell apoptosis.

**Conclusions:**

Our data provide insights into the change of lipidomics landscape in β cells during insulitis and identify a protective mechanism by omega-3 fatty acids.

Video Abstract

**Supplementary Information:**

The online version contains supplementary material available at 10.1186/s12964-023-01437-1.

## Background

Type 1 diabetes (T1D) affects over 1.25 million people in the U.S. and is characterized by the autoimmune destruction of the insulin-producing β cells [1]. The β-cell death disrupts blood glucose homeostasis, leading to health complications that reduce the life expectancy of individuals with the disease by 11 years in males and 13 years in females [2]. T1D treatment relies on exogenous insulin administration, and there is no cure or permanent remission from the disease. Therefore, a better understanding of the mechanisms contributing to β-cell loss is warranted in developing alternative therapies.

T1D onset is associated with changes in serum lipid profiles, including increases in blood triacylglycerol and cholesterol levels, resulting from poor glycemic control [3]. Lipids also contribute to disease development and circulating lipids are biomarkers for autoimmunity and T1D progression at time points prior to the disease onset [4, 5]. Moreover, they are also essential mediators of islet inflammation (insulitis) [6–8]. During insulitis, pro-inflammatory cytokines activate phospholipases, such as 85/88 kDa calcium-independent phospholipase A2 (PLA2G6 or iPLA2β), leading to the cleavage of phosphatidylcholine (PC) into lysophosphatidylcholine (LPC) [7, 9, 10]. This reaction results in the release of fatty acids, such as ω-6, a precursor of prostaglandins, leukotrienes, and thromboxanes that have inflammatory and apoptotic actions, contributing to β-cell apoptosis [11–13]. Conversely, dietary supplementation with ω-3 fatty acids has been shown to reduce the risk of developing islet autoimmunity by 55% in humans and the development of diabetes in non-obese diabetic (NOD) mice by 60% [14, 15]. Despite the latest development in this area, the role of the islet lipidome during the progression of T1D is poorly understood. Mass spectrometry analysis of islets has identified LPC and ceramides as essential molecules in β-cell death [10]. However, deep characterization of the islet lipid composition during insulitis is still missing.

Here, we measured changes in islet/β cell lipid composition in 3 insulitis models: I) human islets II) a human β-cell line, both exposed to the pro-inflammatory cytokines IL-1β and IFN-γ, which induce similar molecular signatures observed in β cells from individuals with T1D [16], and III) in islets from NOD mice. In this study, we used advance lipidomics techniques to unveil the changes and contribution of lipids in insulitis and β-cell death, including mass spectrometry imaging to determine the spatial location of lipids in the islets from humans and mice. Using various molecular tools, we unraveled a mechanism of β-cell death regulation by polyunsaturated fatty acids.

## Methods

### Mice

Female 6-week-old non-obese diabetic NOD/ShiLtJ (#001976) and non-obese diabetes-resistant NOR/LtJ (#002050) mice were purchased from Jackson Labs. The husbandry of mice and experimental procedures were performed according to an approved IACUC protocol at the University of Colorado (#00045). Mice were housed for < 1 week prior to islet isolation. Approximately 320–520 islets (*n =* 3) were isolated using a Histopaque gradient centrifugation and further handpicking of islets as described previously [17].

### Human pancreatic islet and EndoC-βH1 cells

The lipidomics and proteomic data of the human islets (*n =* 10, deidentified tissue were obtained from the Integrated Islet Distribution Program, details in Table S1) and EndoC-βH1 cells (*n =* 3) treated with 50 U/mL IL-1β + 1000 U/mL IFN-γ (cytokine cocktail 1 or CT1) for varying hours were collected as described in detail elsewhere [18, 19].

### MIN6 cell line culture and treatment

MIN6 cells were a gift from the Yamagata lab, and were cultured in DMEM containing 4.5 g/L each of D-glucose and L-glutamine, 10% FBS, 100 units/mL penicillin, 100 μg/mL streptomycin and 50 mM 2-mercaptoethanol maintained at 37 °C in a 5% CO_2_ atmosphere [20, 21]. For knockdown experiments, cells were transfected using Lipofectamine RNAiMAX (Invitrogen) with SMARTpool ON-TARGETplus non-targeting siRNA (Dharmacon, cat#D-001810-10-20) or siRNA targeting *Pla2g6*, *Adprhl2* (Dharmacon, cat# L-051819-01-0020)*,* and *Suz12* (Dharmacon, cat# L-040180-00-0005) for varying hours, followed by cytokine cocktail 2 (CT2: 100 ng/mL IFN-γ: R&D, cat#485-MI-100, 10 ng/mL TNF-α: R&D, Cat#410-MT-010, and 5 ng/mL IL-1β: R&D, cat #401-ML-005) treatment for 24 h. Cells were treated at 80% confluency with 80 μM arachidonic acid (Cayman, CAS#506–32-1), linoleic acid (Cayman, CAS#60–33-3), eicosapentaenoic acid (Cayman, CAS#10417–94-4), or docosahexaenoic acid (Cayman, CAS#6217–54–5) or equal volume of 100% ethanol (vehicle control) in combination with CT2 for 8 hrs.

### Lipidomic analysis

Samples were subjected to metabolite, protein, and lipid extraction (MPLEx) with all the procedures done on ice or 4 °C to reduce sample degradation, as previously described [22]. Extracted lipids were dissolved in methanol and loaded on a reversed-phase column connected to a NanoAcquity UPLC system (Waters) and interfaced with a Velos Orbitrap mass spectrometer (Thermo Fisher Scientific) [23] (see Table S2 for parameters). Lipid species were identified using LIQUID [23] and manually validated based on the retention time, precursor isotopic profile, diagnostic fragments from head groups and fatty acyl chains. Isomers were named in alphabetical order based on their elution times. Features of identified lipids were extracted with MZmine [24]. Peaks were detected with a 20% intensity tolerance, noise level of 5e3, mass tolerance of 0.008 m/z and retention time tolerance of 0.3 min. Chromatograms were built with a time span of 0.1 min, minimum height of 5 × 10^3^ and mass tolerance of 0.008 m/z. Peaks were then aligned with joint aligner with mass tolerance of 0.01 m/z, retention time tolerance of 0.2 min, and weights of both retention time and m/z of 1. Missing values were filled with gap filling based on the same retention time and mass tolerance of 0.008 m/z. Statistics were performed using standard paired *t-test* followed by a multiple-test Bonferroni correction.

### Proteomic analysis

MIN6 cell proteins were dissolved in 50 mM NH_4_HCO_3_, 8 M urea and 10 mM dithiothreitol and incubated for 1 h at 37 °C with 800 rpm shaking. Then 400 mM iodoacetamide was added to a final concentration of 40 mM, and the mixture was incubated for another hour in the dark at room temperature. The reaction mixture was 8-fold diluted with 50 mM NH_4_HCO_3_, and 1 M CaCl_2_ was added to a final concentration of 1 mM. Proteins were digested for 3 h at 37 °C using trypsin at 1:50 enzyme: protein ratio. Digested peptides were desalted by solid-phase extraction using C18 cartridges (Discovery, 50 mg, Sulpelco) and dried in a vacuum centrifuge. Peptides were analyzed on a Waters NanoAquity UPLC system coupled with a Q-Exactive mass spectrometer (see Table S2 for parameters). Data were processed with MaxQuant software (v.1.5.5.) [25] using the mouse reference proteome database from UniProt Knowledge Base (downloaded on August 14, 2018). Protein N-terminal acetylation and oxidation of methionine were set as variable modifications, and cysteine carbamidomethylation as fixed modification. Mass shift tolerance was used as the default setting of the software. Only fully tryptic-digested peptides were considered, allowing up to two missed cleaved sites per peptide. Quantification of proteins was done using the intensity-based absolute quantification (iBAQ) method [26]. Data were log2 transformed and normalized by linear regression and central tendency using InfernoRDN (formerly Dante) [27]. Statistically significant proteins were determined by ANOVA or by Student’s *t*-test.

### Bioinformatics analysis

The significantly different lipids and proteins were submitted to ontology/function-enrichment analysis using Lipid MiniOn [28] and DAVID [29] tools, respectively. For the Lipid MiniOn analysis, the full list of identified lipids was set as the background and the significantly different species as the query. Ontologies were considered enriched with a *p* ≤ 0.05 using the Fisher’s exact test. For the DAVID analysis, the differentially abundant proteins were set as the query and the entire genome was set as the background. Only enriched pathways (*p* ≤ 0.05) of the KEGG database were used and they were grouped based on shared proteins using Enrichment Map [30].

### Mass spectrometry imaging analysis

Nanospray desorption electrospray ionization (nano-DESI) mass spectrometry imaging was performed on a Q Exactive HF-X mass spectrometer (Thermo Fisher Scientific) [31]. High-resolution nano-DESI probes were assembled using two fused silica capillaries pulled to O.D. 15–25 μm. A shear force probe with a tip diameter of ~ 10 μm was integrated with the nano-DESI probe and was used to precisely control the distance between the probe and the sample. The position of the samples was controlled by a motorized XYZ stage. Samples were scanned at a rate of 10 μm/s under the nano-DESI probe in lines with a step of 20 μm between the lines. A 9/1 (v/v) methanol/water mixture containing 200 nM LPC 19:0 (internal standard) was propelled through the nano-DESI probe at 500 nL/min (see Table S2 for parameters). A custom-designed software, MSI QuickView, was used for data visualization and processing. Ion images were generated by normalizing the signal of the analyte to the signal of the internal standard (LPC 19:0). Lipids were putatively identified by matching based on the high mass accuracy against the species characterized in the lipidomics analysis.

### Fluorescence in-situ hybridization (FISH)

For FISH experiments, ten oligonucleotide probes containing targeting and 3′ readout overhang domains were designed against the *Pla2g6* transcript coding region (sequences in Table S3) [32]. Targeting domains were 20-nt long, complementary to the *Pla2g6* mRNA, with 40–60% CG content and without self-repeats or inner loop structures. A secondary probe labeled with two Alexa647 molecules was used to hybridize with the 3′ overhang domain. Samples were fixed with fresh 4% paraformaldehyde. After quenching the residual paraformaldehyde with 0.1% sodium borohydride, samples were permeabilized with 0.2% Triton-X 100 and stored in 70% ethanol. The primary and secondary probes (50 nM final concentration) and anti-insulin antibody (Table S3) (1000-fold dilution) were diluted in hybridization solution (10% dextran sulfate, 15% formamide, 1× SSC, 3.4 mg/mL tRNA, 0.2 mg/mL RNase-free BSA, 2 mM ribonucleoside vanadyl complex). Samples were incubated with probes overnight at 37 °C overnight in a humid chamber. Samples were rinsed with 15% formamide in 1× SSC, followed by staining with Atto 488-conjugated secondary antibody (Table S3) and DAPI. Images were collected on an Olympus IX71-based single-molecule microscope equipped with 405 nm, 488 nm, and 640 nm solid lasers, 100× oil immersion objective lens (NA 1.4) and an EMCCD camera (Andor iXon Ultra 897). Fluctuation localization imaging-based FISH (fliFISH) was used to extract the location of *Pla2g6* mRNA in tissue sections [32]. Photoswitching was activated by using GLOX-containing buffer: 50 mM Tris, 10 mM NaCl, 10% glucose, 560 μg/mL glucose oxidase, 34 μg/mL catalase and 1% β-mercaptoethanol.

### Tissue processing and immunohistochemistry

Mouse pancreata were dissected, washed in ice-cold PBS, and fixed for 4 h in 4% paraformaldehyde at 4°C. The tissues were then incubated in 30% sucrose overnight and frozen at optimal cutting temperature. Cryosections with 10 μm thickness were obtained and immunohistochemistry for PLA2G6 was performed using the Mouse-on-Mouse kit (Vector Labs) and the Vectastain ABC HRP kit (Vector Labs).

### Quantitative real-time PCR analysis

Cells were harvested using Tri reagent (Zymo, Cat#R2050–1-200) and total mRNA was extracted using the RNA Clean & Concentrator™-5 (Zymo, Cat# R1014) RNeasy Mini kit (Qiagen). RNA was quantified using nanodrop and was aliquoted accordingly to perform one-step qRT-PCR using QuantiNova™ SYBR Green RT-PCR Kit (Qiagen, Cat. No. / ID: 208154). Pre-designed mouse primers were ordered from Millipore sigma (Cat# KSPQ12012) and their efficiency was validated prior to use (oligonucleotide information in Table S3). Best housekeeping gene for our experiment was determined using BioRad Reference 12 gene panel (Cat# 10025216). The 3 best housekeeping genes with the least standard deviation among the tested groups were selected to normalize the expression (Nono, Rpl13a, and Hprt). Expression level of genes were calculated using Livak’s method [33].

### Western blotting

Cells were harvested in RIPA buffer or M-PER™ Mammalian Protein Extraction (Thermo, Cat#78501) containing protease and phosphatase inhibitors (Thermo, Cat#A32959). Protein quantity was measured using BCA assay and was prepared using 4x NuPAGE LDS sample buffer accordingly. Samples were run on a Bis-Tris 4–12% Mini Gels and proteins were transferred to PVDF membranes. After blocking in 5% milk in TBS containing 0.1% Tween 20 or StartingBlock™ T20 (TBS) Blocking Buffer (Thermo, Cat#37543), membranes were incubated in specific primary antibodies at 4°C overnight. Anti-mouse and anti-rabbit horseradish peroxidase-conjugated antibodies (Table S3) were used for secondary antibodies and enhanced chemiluminescent substrate was used for signal detection.

### Apoptotic assay

The experiment was done as per manufactures protocol (Promega Cat# G8092). Briefly, MIN6 cells were treated with EPA and DHA for 48 h followed by 24 h cytokine cocktail CT2 treatment. Appropriate amount of caspase-Glo 3/7 reagent was added to the wells. The contents were gently mixed for 30 s and luminescence was read for 3 h every 30 min interval. The time point with the highest signal was selected for analysis.

### Data visualization and statistical analysis

Basic data analysis was done in Microsoft Excel. All statistical analysis and data visualization (volcano plots and bar graphs) were performed using GraphPad Prism 9 (Version 9.4.1). Heatmap was plotted using Perseus-MaxQuant [34]. Protein pathway analysis was visualized using cytoscape_v3.9.1. ChIP-Seq data visualization was performed using IGV software.

## Results

### Lipidome analysis of insulitis models

We studied three models of insulitis: (I) human EndoC-βH1 cells exposed to the cytokine cocktail 1 (CT1: IL-1β + IFN-γ) for 48 h, (II) human islets exposed to CT1 for 24 h, and (III) islets from NOD mice at the pre-diabetic stage (6-week-old) vs. age-matched NOR mice. To verify that 6 weeks of age corresponds to the initial T1D developmental stages of NOD mice, we performed proteomics analysis of islets from both NOD and NOR mice (Tables S4–S5) and compared the results against published proteomics data of EndoC-βH1 cells and human islets exposed CT1 [18, 19]. We observed an upregulation of inflammatory markers, such as the antigen transport protein Tap1, the transcription factor STAT1 and the interferon-induced guanylate-binding protein GBP2 (Fig. S1). None of the samples had reduced levels of insulin (Fig. S1), confirming that the islets from NOD mice had inflammation but remained in a pre-diabetic stage without significant β-cell loss.

**Fig. 1:**
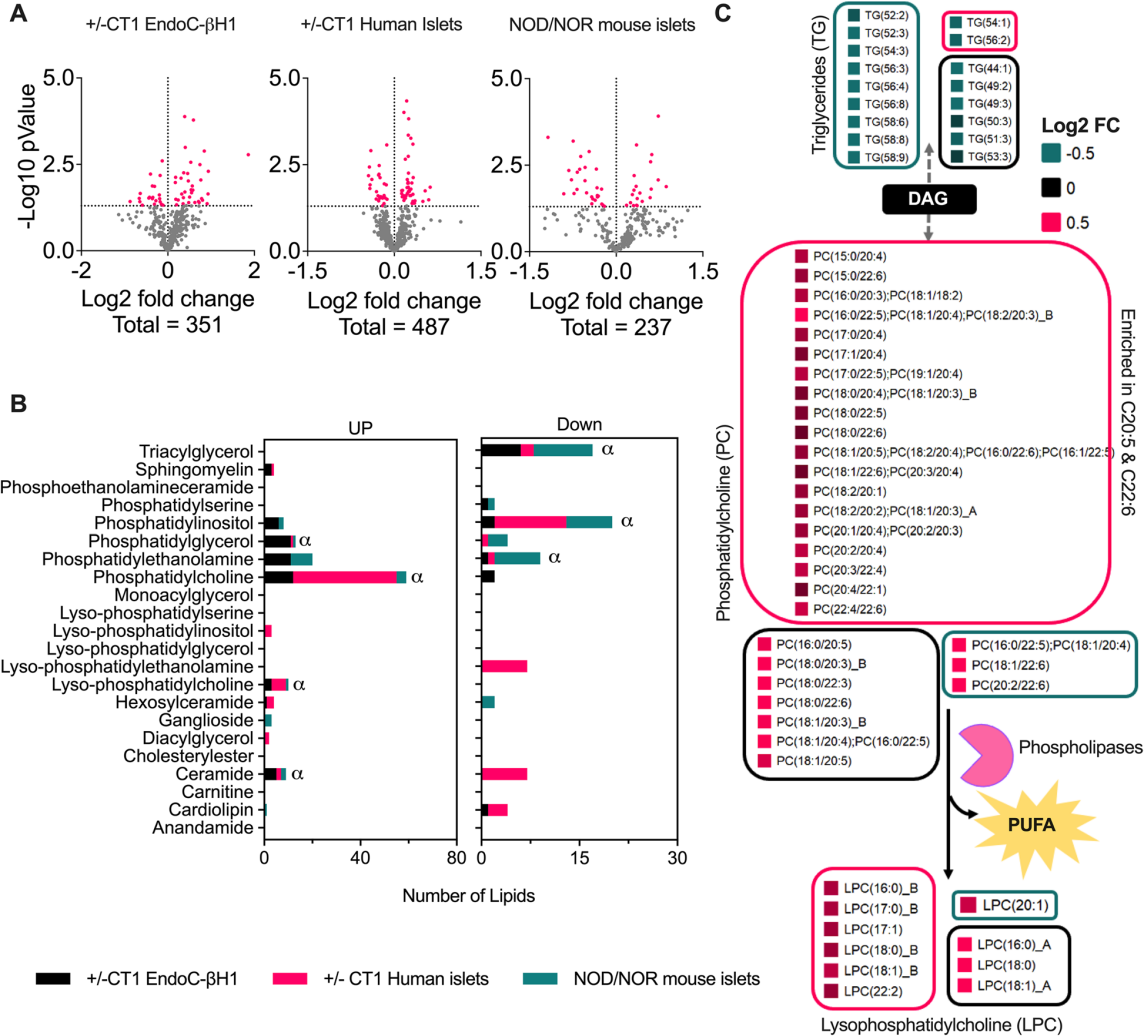
Global lipidomic analysis of 3 common insulitis models, i.e., EndoC-βH1 (*n =* 3) cells and human islets (*n =* 10) exposed to CT1 (IL-1β and INF-γ) for 48 h and islets from non-obese diabetic (NOD) mice in pre-diabetic stage (6 weeks of age) vs. age-matched NOR mice (*n =* 3). Lipids were extracted and analyzed by liquid chromatography-tandem mass spectrometry. **a** Volcano plots of the lipid species relative abundances. **b** Number of lipid species significantly (Student’s *t*-test *p ≤* 0.05) regulated in each class. “α” represents common lipid species upregulated or downregulated in all three insulitis models. **c** Lipid species that are consistently regulated in the 3 insulitis models. Each lipid species is named with the abbreviation of its class (e.g., LPC and PC) followed by the length of the fatty acid and number of double bonds (separated by a colon) in parenthesis. The letters after the lipid names represent different isomers that are separated in the chromatography in alphabetical order. The relative abundance in T1D model vs. control is color-coded. Isobaric coeluting species (separated by semicolons) were co-quantified

Next, lipidomics analysis of the insulitis models – EndoC-βH1 cells (+/− CT1) human islets (+/− CT1) and murine islets (NOD vs. NOR), resulted in the identification and quantification of 369, 558 and 251 lipid species, respectively (Fig. 1a, Tables S6–S11). The overall profiles of many regulated lipid classes were different comparing human islets with EndoC-βH1 cells and murine islets insulitis models (Fig. 1b). However, certain lipid groups including LPC and PC with long polyunsaturated fatty acyl chains were commonly increased across all three models, whereas triglycerides (TGs) were consistently decreased (Fig. 1b). To better understand commonly regulated lipid metabolic processes across all models, we performed an enrichment analysis using Lipid Mini-On [28]. This tool determines whether any specific lipid feature, such as subclass, fatty acyl chain length, or degree of unsaturation, is enriched among the differentially regulated lipids. Polyunsaturated fatty acids C22:5 and C22:6 were enriched among the 3 classes of commonly regulated lipids across the insulitis models (Fig. 1c). We hypothesized that lipid groups commonly regulated in EndoC-βH1 cells, human islets, and murine islets in response to pro-inflammatory cytokines were important for T1D pathogenesis; therefore, we further investigated them.

### Spatial distribution of lipids and lipids metabolizing enzyme in islets

**Fig. 2:**
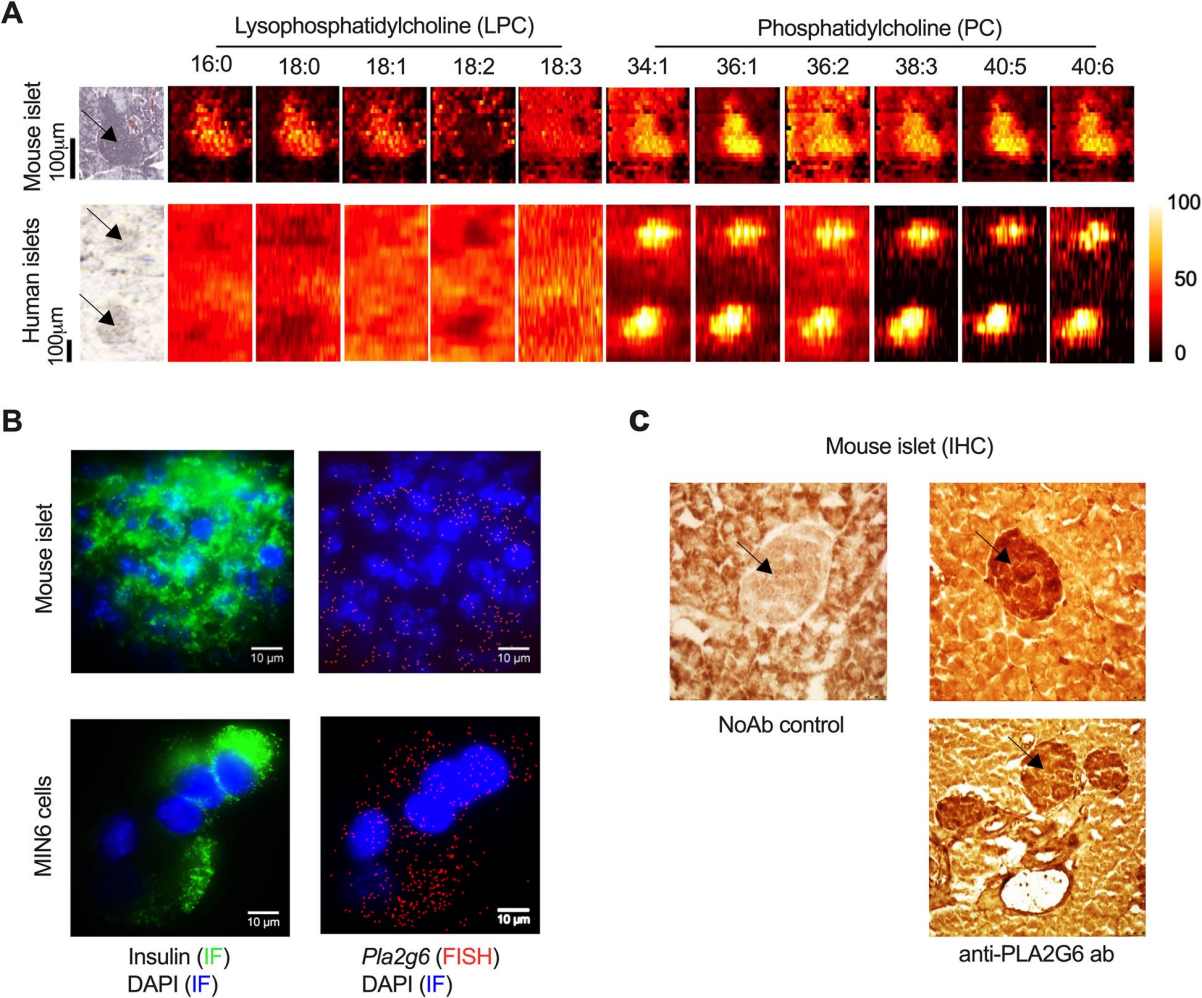
Spatial localization of lysophosphatidylcholines, phosphatidylcholines and phospholipase PLA2G6 in pancreata. **a** Chemical image of mouse and human pancreata by mass spectrometry. Each image shows either the optical image or color-coded distribution of different lipids. Lipid species were identified by matching against the lipids characterized and quantified on the lipidomics analysis based on their accurate masses. **b** PLA2G6 fluorescence in situ hybridization (FISH) of islets from non-obese diabetes resistant (NOR) mice (6 weeks of age) and MIN6 cell line. Cells and tissues were stained with anti-insulin antibody (green), DNA stain 4′,6-diamidino-2-phenylindole (DAPI – blue) and fluorescent-labeled antisense Pla2g6 oligonucleotide (red). **c** Immunohistochemistry (IHC) analysis of PLA2G6. Tissue was stained with biotin-conjugated anti-Pla2g6 antibodies followed by avidin-conjugated horseradish peroxidase. Localization was visualized by horseradish peroxidase-mediated oxidation and precipitation of 3,3′-diaminobenzidine (brown). The images are representative of two independent experiments

The spatial distribution of PCs and LPCs were determined in murine and human pancreata using mass spectrometry imaging. Lipid molecular species were putatively identified by matching their measured m/z against the calculated m/z of characterized species from the lipidomics analyses in Fig. 1. PC species with (PC(38:3), PC(40:5) and PC(40:6)) and without (PC(34:1), PC(36:1), PC(36:2)) longer/polyunsaturated fatty acyl (PUFA) chains and LPC species (LPC(16:0), LPC(18:0) and LPC(18:1)) were enriched in mouse islets (endocrine tissue) compared to the surrounding exocrine tissue. In human islets, an enrichment of PC but not of LPC species was observed compared to the surrounding tissue (Fig. 2a). As the solvent composition used in imaging experiments prevented an efficient extraction of the TGs, we were unable to determine the localization of that lipid class. Nevertheless, we were able to confirm the spatial localization of PC species with long polyunsaturated fatty acids and LPC in the islets.

We also investigated the distribution of the phospholipase PLA2G6, which hydrolyses PCs into LPCs and fatty acids. These fatty acids include polyunsaturated ones that have been shown to contribute to the development of T1D [7]. Consistent with the localization of PCs and LPCs, PLA2G6 was also enriched, as demonstrated by combining fluorescent in situ hybridization (mouse islets and MIN6 cells) and immunohistochemistry (mouse islets) (Fig. 2b, c). Insulin immunofluorescence was used as a marker for β cells (Fig. 2b). Taken together, our data support the notion that the generation of LPCs is mediated by phospholipases, such as PLA2G6, which is enriched in islets compared to the surrounding tissue.

### PLA2G6-dependent cytokine regulation in MIN6 cells

**Fig. 3:**
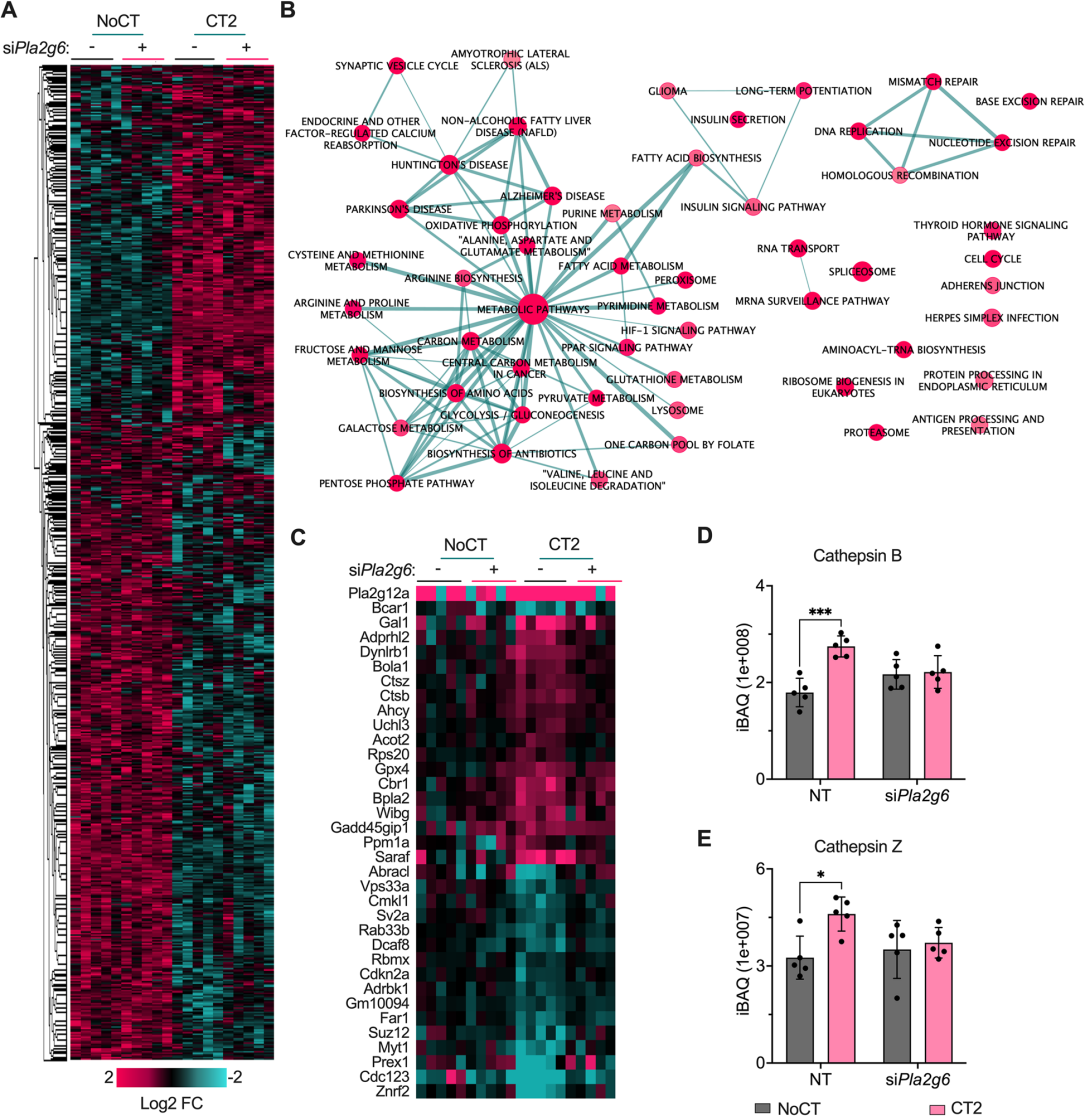
Pro-inflammatory cytokines and Pla2g6-dependent proteome remodeling in MIN6 cells. **a** Cytokine cocktail CT2 (IL-1β + IFN-γ + TNFα)-dependent protein expression in *Pla2g6* siRNA (si*Pla2g6*) MIN6 cells (*n =* 5, +SD). Nontarget siRNA was used as a transfection control. **b** KEGG pathways enriched with proteins differentially abundant in IL-1β + IFN-γ-treated MIN6 cells. Pathways were grouped based on shared proteins using Enrichment Map tool in Cytoscape [30]. Each pathway is represented by a node, and their degree of connectivity (thickness of the edges) is proportional to the number of shared proteins between the pathways. **c** Pla2g6-dependent protein abundance changes of CT2-treated MIN6 cells. Abundance profiles of cathepsin Z (**d**), cathepsin B (**e**). Statistical test: ** *p* ≤ 0.01 and *** *p ≤* 0.001 by 2way-ANOVA and “Šídák’s multiple comparisons test

PLA2G6-mediated cleavage of PC results in generation of LPC and fatty acids, many with distinct biological activities, such as ω-6 and ω-3 FAs. Therefore, we studied the PLA2G6-dependent cytokine signaling landscape in mouse MIN6 cells. For this, we performed global proteomics of untreated control (NoCT) cells (nontargeting vs. *Pla2g6* siRNA) treated with cytokine cocktail 2 (CT2: IL-1β + INF-γ + TNFα) for 24 h. We observed that the CT2 treatment led to changes in abundance of 1043 out of the 5212 identified and quantified proteins (Fig. 3a, Table S12). A functional enrichment analysis of the differentially regulated proteins revealed that 52 KEGG pathways were regulated by the cytokine treatment (Fig. 3b). Only a small fraction of the cytokine-regulated proteins, 35 proteins, were depended on PLA2G6 (Fig. 3c, Table S13). Among these proteins, the expression of cathepsin Z and cathepsin B, lysosome protease marker, was significantly upregulated with the CT2 treatment, but this upregulation was abolished in the *Pla2g6* siRNA group (Fig. 3d, e). Overall, the analysis showed a strong regulation of the MIN6 cell proteome by cytokines, of which a small subset of proteins depended on PLA2G6.

### Regulation of poly(ADP)ribosylation proteins by PLA2G6

**Fig. 4:**
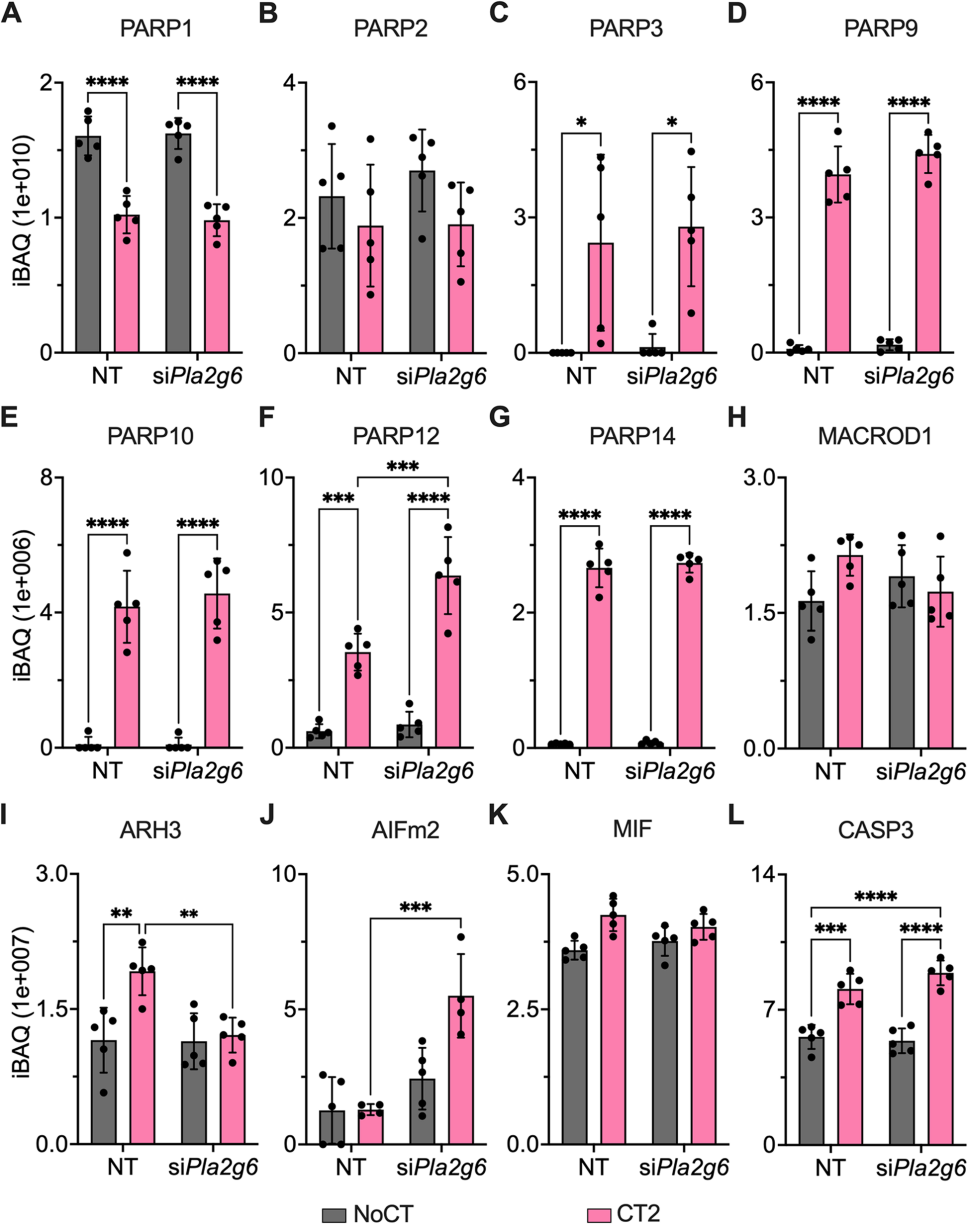
Regulation of ADP-ribosylation enzymes by pro-inflammatory cytokines and Phospholipase enzyme. **A**-**K** Cytokine cocktail CT2 (IL-1β + IFN-γ + TNFα)-dependent protein expression of ADP-ribosylation enzymes and PARP-mediated apoptosis markers in *Pla2g6* siRNA (si*Pla2g6*) MIN6 cells (*n =* 5, +SD). Statistical test: * *p* ≤ 0.05, ** *p* ≤ 0.01, and *** *p ≤* 0.001 by 2wayANOVA and “Šídák’s multiple comparisons test

Among the PLA2G6-dependent cytokine-regulated proteins, ADP-ribosylhydrolase ARH3 (*Adprhl2* gene) removes ADP-ribosylation from proteins. ADP-ribosylation induces β-cell death in mice [35]. Therefore, we investigated the abundance profiles of other enzymes involved in ADP-ribosylation within the proteomics data: PAR polymerases (PARP), PAR glycohydrolases (PARG), MacroD1, MacroD2, terminal ADP-ribose protein glycohydrolase 1 (TARG1) and ADP-ribosylhydrolases (ARHs). PARP1 abundance significantly decreased by approximately 35% in both *Pla2g6* and nontargeting siRNA groups when treated with CT, whereas PARP2 levels were not affected (Fig. 4a, b). Conversely, PARP3, − 9, − 10, − 12, and − 14 were strongly upregulated (> 5 fold) when cells were treated with CT2 in both *Pla2g6* and nontargeting siRNA groups (Fig. 4c-g). Among the PAR hydrolases, only ARH3 and MacroD1 were detected, showing a statistical increase in abundance of ARH3 by 66% and no statistically significant change in MacroD1 with CT2 treatment (Fig. 4h, i). Specifically, ARH3 levels were statistically reduced in *Pla2g6* siRNA compared to CT2-nontargeting siRNA group, indicating a regulation of ARH3 by PLA2G6 (Fig. 4i). Concurrently, we investigated the effect of PLA2G6 on the PARP-regulated apoptosis, by assessing the abundance of apoptosis-inducing factor (AIFm2), macrophage migration inhibitory factor (MIF) and caspase 3 levels [36, 37]. We observed that AIFm2 levels in the CT2-*Pla2g6* siRNA group statistically increased by 62% compared to CT2, but no change in the abundance of MIF, the binding partner of AIF, was observed. The total caspase 3 level was significantly high post-CT2 treatment, which further showed an increased trend of 22% in the absence of PLA2G6 (Fig. 4j-l). This, in combination with a reduced level of ARH3 in CT2-siPLA2G6 group, indicates PARP-mediated apoptosis in β cells.

**Fig. 5:**
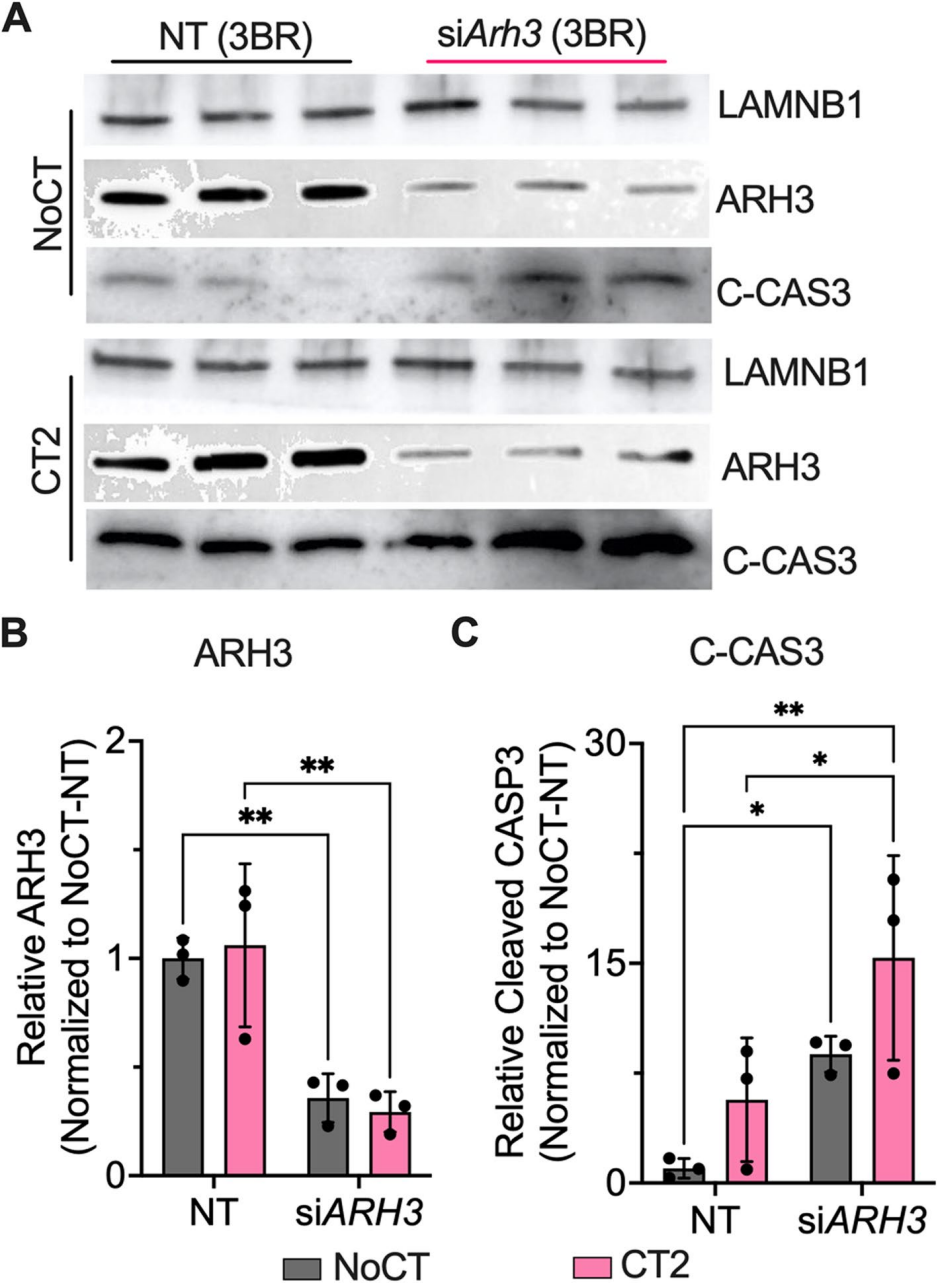
ARH3 regulates cytokine-mediated β-cell apoptosis. Western blot analysis of *ARH3* siRNA (si*ARH3*) MIN6 cells treated with cytokines (**a**) (BR: Biological replicates). **b**, **c** relative levels of ARH3 (**b**) and cleaved caspase 3 (**c**) bands normalized to LAMINB1. To ensure reproducibility, we performed 3 independent experiments. Statistical test: * *p* ≤ 0.05, ** *p* ≤ 0.01, and *** *p ≤* 0.001 by 2wayANOVA and “Šídák’s multiple comparisons test

We tested the effects of ARH3 in cytokine-mediated apoptosis by knocking down *Adprhl2* (*Arh3 siRNA*) and performed western blots for cleaved caspase 3, as a marker of apoptosis. A 65–70% knockdown efficiency was obtained in *Arh3* siRNA cells (Fig. 5a, b). The CT2 treatment significantly increased the expression of cleaved caspase 3 by ~ 8.8-fold in the nontargeting siRNA compared to the NoCT group (Fig. 5c). In the *Arh3* siRNA group, the basal level of cleaved caspase 3 was high, but the levels significantly increased further by 1.75-fold upon CT2 treatment compared CT-NT group, confirming the protective function of ARH3 against cytokine-induced mouse β-cells death.

### Regulation of Arh3 expression by SUZ12 through lipid signaling

**Fig. 6:**
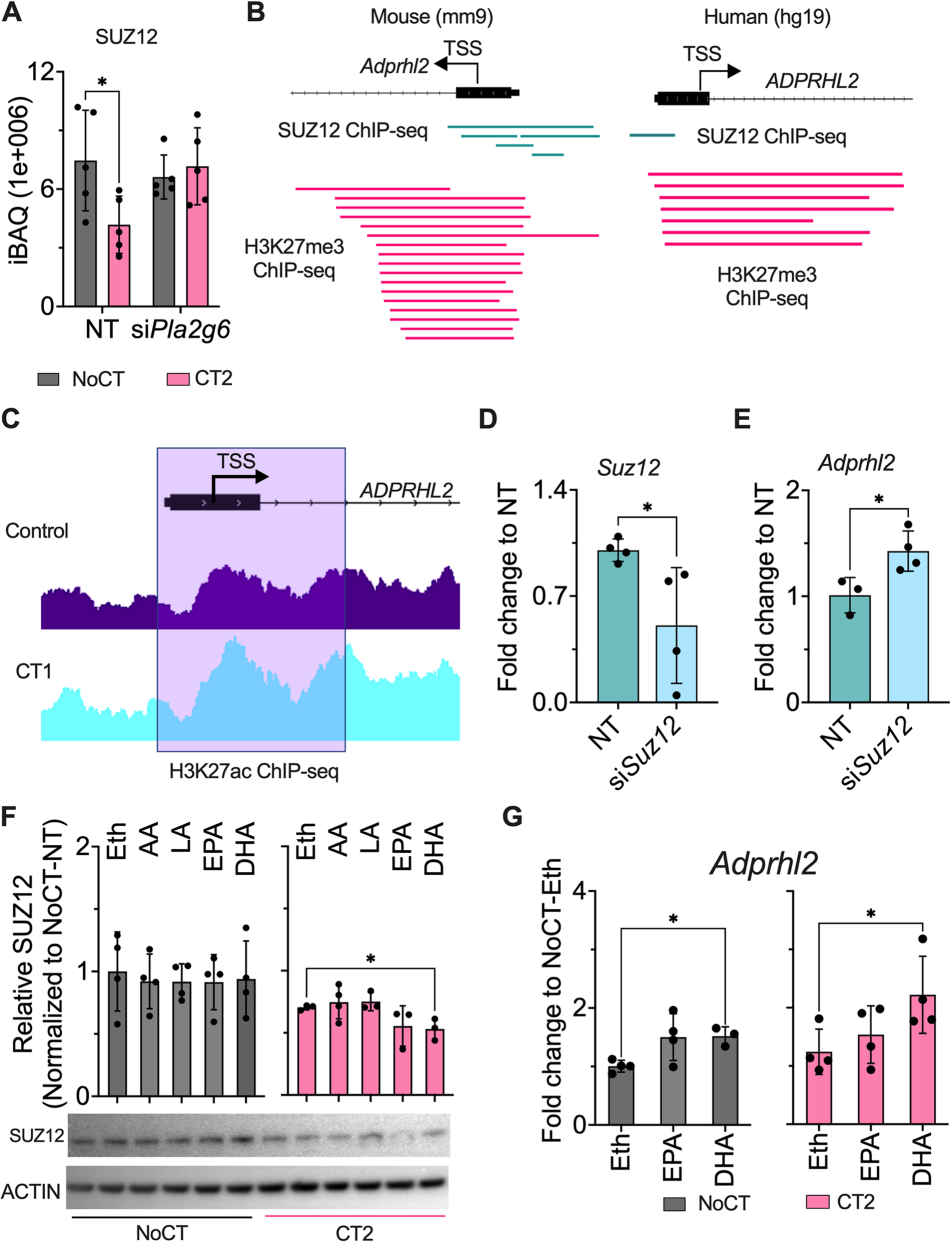
Regulation of the ARH3 gene *Adprhl2* expression by ω-3 fatty acids and SUZ12. **a** SUZ12 protein expression in *Pla2g6* siRNA (si*Pla2g6*) MIN6 cells with cytokine cocktail CT2 (IL-1β + IFN-γ + TNFα) treatment (*n =* 5, +SD). **b** ChIPseq mouse (mm9) and human (hg19) data were retrieved from ChIP-atlas database (https://chip-atlas.org/peak_browser). The individual line represents independent studies reporting enrichment of SUZ12 (Green) and H3K27me3 (Pink) at *ADPRHL2* transcriptional start site (TSS). **c** Representative western blot image and relative level of SUZ12 protein normalized to Actin post CT2 and ω-6 (arachidonic acid - AA & linoleic acid - LA) and ω-3 (eicosapentaenoic acid - EPA & docosahexaenoic acid - DHA) fatty acid treatment (*n =* 3–4, +SD). Ethanol (Eth) was used as solvent control for the fatty acids (FAs). **d** *Adprhl2* mRNA expression post-CT2 and ω-3 FA (EPA and DHA) treatment (*n =* 3–4, +SD). **e**, **f** *Suz12* and *Adprhl2* mRNA expression in Min6 cells with *Suz12* siRNA (si*Suz12*) (*n =* 3–4, +SD). **g** Re-analyzed H3K27ac ChIPseq data of CT1 (IL-1β + IFN-γ) treated Human islets [19]. *p ≤ 0.05 for A was calculated by 2wayANOVA and Šídák’s multiple comparison test, for C students’ *t*-test, D one-way ANOVA followed by Šídák’s multiple comparisons test, and for F & G, students’ *t*-test was used. Specifically, the normality and outlier test for the molecular experiment were tested using “The shapiro-Wilk test” and Dixon’s test, with a threshold of *p* < 0.2, respectively

In our proteomic analysis, the level of SUZ12, a component of histone methyltransferase polycomb complex 2 (PRC2), was statistically decreased in the CT2-treated group but not in CT2-si*PLA2G6*, indicating SUZ12 is regulated in a PLA2G6-dependent manner (Fig. 6a). As SUZ12 and by extension, PRC2 is a known gene suppressor, we investigated the role of SUZ12 in the PLA2G6-ARH3 signaling pathway.

The ChIP-atlas database showed the binding of SUZ12 and enrichment of histone H3K27 trimethylation at the transcription start site of *Adprhl2* gene in both mice and humans (Fig. 6b). Reanalysis of our previously published H3K27ac ChIP data from CT1-treated human islets indicated a minute enrichment of H3K27ac at the *Adprhl2* transcription start site (Fig. 6c) [19]. To test if a decrease in SUZ12 was responsible for enhancing *Adprhl2* expression, we knockdown *Suz12* and assayed its and *Adprhl2* expression by qPCR. The knockdown experiment significantly reduced the expression of *Suz12* by 50% and increased *Adprhl2* expression by 40% (Fig. 6d, e).

Dimri et al. showed that ω-3 fatty acids, such as docosahexaenoic and eicosapentaenoic acids, cause the degradation of SUZ12 and consequently enhance the expression of PRC2’s target genes [38]. As PLA2G6 can cleave PC-PUFA into LPCs and ω-3 fatty acids, we hypothesized that docosahexaenoic and eicosapentaenoic acids produced upon cytokine-mediated activation of PLA2G6 causes degradation of SUZ12, enhancing ARH3 expression by reducing trimethylation at the *Adprhl2* transcription start site. To test this hypothesis, we treated MIN6 cells with docosahexaenoic and eicosapentaenoic acids in the presence or absence of CT2 for 8 h. Western blot analysis showed that docosahexaenoic acid significantly reduces the abundance of SUZ12 by 25% in the presence of CT2 (Fig. 6f). Concomitantly, *Adprhl2* expression increased by 50% when treated with docosahexaenoic acid in a similar trend with CT2 treatment or docosahexaenoic acid /CT2 combined (Fig. 6g). These results unraveled an intricate lipid signaling mechanism that increases ARH3 protein level in β cells to protect them against cytokine-mediated apoptosis via ω-3 fatty acid-induced SUZ12 downregulation, which in turn causes de-repression of the *Adprhl2* gene.

### Effect of omega 3 fatty acids on MIN6 cells

**Fig. 7:**
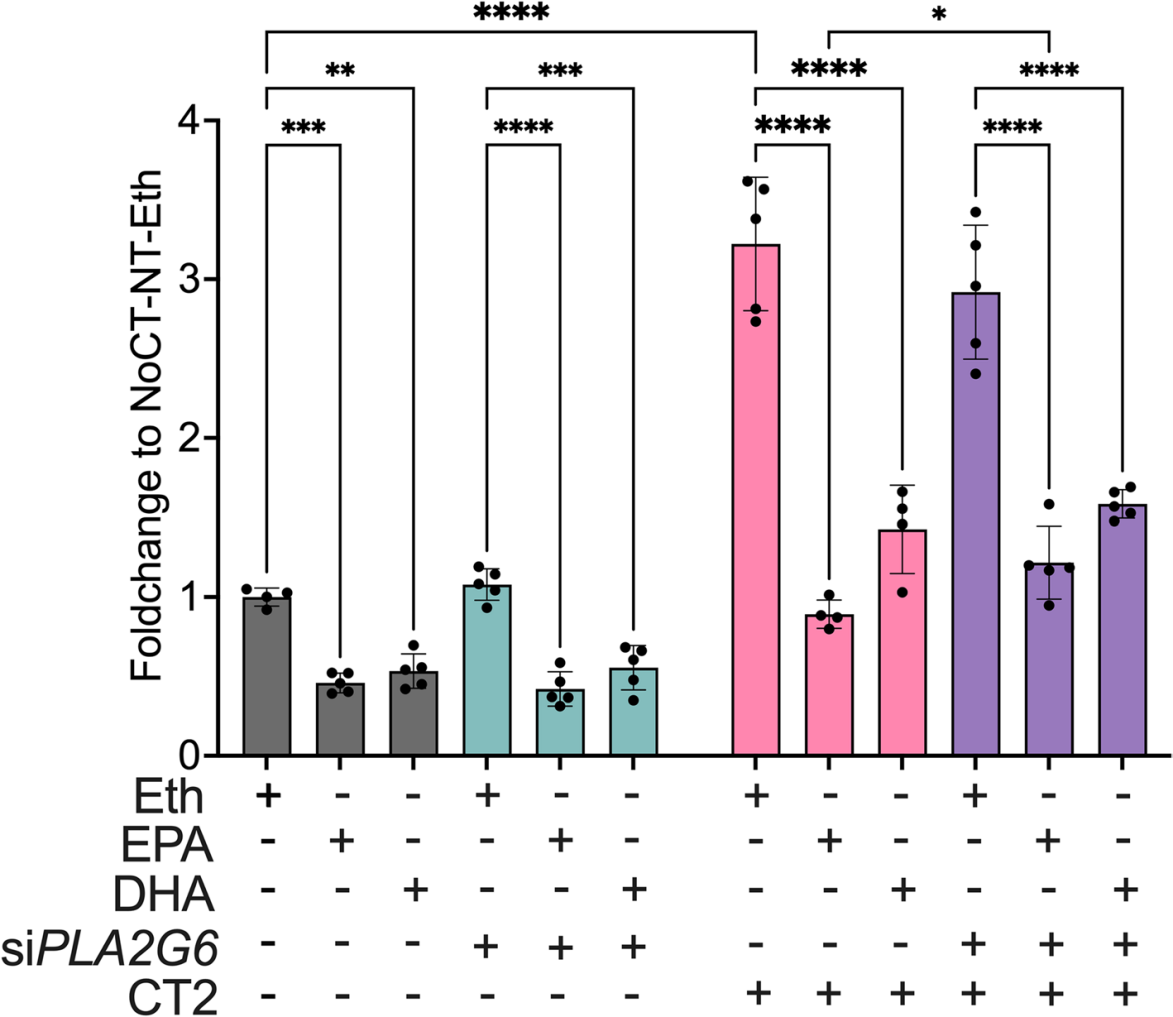
Protective effect of ω-3 fatty acids against cytokine-induced apoptosis. Apoptosis was measured by caspase3/7 activity in MIN6 cells treated with ω-3 fatty acids or ethanol (Eth) and PLA2G6 siRNA (si*PLA2G6*) for 48 h followed by 24 h of cytokine cocktail CT2 (IL-1β + IFN-γ + TNFα) treatment (*n =* 4, +SD). *p ≤ 0.05, **p ≤ 0.01, *** *p ≤* 0.001 and ****p ≤ 0.0001 by 2wayANOVA and “Uncorrected Fisher’s LSD” test. Specifically, the normality and outlier test for the molecular experiment were tested using “The shapiro-Wilk test” and Dixon’s test, with a threshold of *p* < 0.2, respectively

To test the effect of ω-3 fatty acids on cell apoptosis, we performed a caspase 3/7 assay in cytokine-treated parental and *Pla2g6* RNAi MIN6 cells pre-treated with docosahexaenoic and eicosapentaenoic acids. Cells were incubated with docosahexaenoic and eicosapentaenoic acids for 48 h and then treated for 24 h with CT2. Eicosapentaenoic acid reduced CT2-mediated apoptosis by 75%, whereas docosahexaenoic acid reduced it by 56% (Fig. 7a). This reduction was partially dependent on PLA2G6, as the eicosapentaenoic acid treatment only reduced apoptosis of *Pla2g6* RNAi by 60%, which is significantly less than in the parental cells (Fig. 7a). Similar trend was observed for the docosahexaenoic acid treatment, although it did not reach significance (Fig. 7a). These results show that ω-3 fatty acids protect cells against pro-inflammatory cytokine-mediated apoptosis and that this protection is at least partially dependent on PLA2G6.

## Discussion

We investigated the remodeling of islet and β-cell lipid composition in response to inflammatory mediators relevant to the pathogenesis of T1D. To increase the confidence of our findings, we performed lipidomic analysis across three insulitis models: EndoC-βH1 cells and human islets exposed to the cytokines IL-1β + INF-γ, and islets from NOD mice at the pre-diabetic stage. This analysis identified key common changes in lipid composition: downregulation of TGs and upregulation of PCs and LPCs with long unsaturated fatty acid chains. Similar observation regarding TGs was reported previously by Oresic et al., and Sorenson et al. [39, 40], however, certain PCs and LPC species were observed to be decreased. It is worth noting that, both studies were done using serum/plasma from humans at different stages of the disease, which differs from our experimental model. TGs are decreased in rat β cells by CT2, likely to accommodate the increased energetic demands [41]. Also, increases in cellular TG content have been associated with protection of rat β cells against the cytotoxic effects of saturated free fatty acids [42]. However, deeper investigations are needed for a better understanding the roles of TGs in T1D development.

The relative increase of PCs with PUFA might be due to an increase in fatty acid oxidation and turnover induced by cytokines, as has been shown in muscles [43] combined with a slower turnover of very long and unsaturated fatty acids [44]. PUFAs are not readily metabolized to supply energetic demands as their degradation requires additional metabolic steps compared to shorter saturated fatty acids [45]. The increase of LPCs in insulitis is due to the activity of phospholipases, such as the PLA2G6, on their precursors, phosphatidylcholines, which has been shown to play a role in the β-cell death [43]. This reaction also releases PUFAs, which are associated with both protection [46] and apoptosis of β cells in response pro-inflammatory cytokines [43]. ω-6 PUFAs are known precursors of immunomediators, such as prostaglandins, leukotrienes, and thromboxanes [47]. Some of these immunomediators have anti-inflammatory properties, while others are known to induce inflammation and apoptosis. For instance, processing of arachidonic acid into 12-HETE by 12-lipoxygenase causes β-cell death [48]. This release of the arachidonic acid from the β-cell membrane induced by cytokines is catalyzed by PLA2G6 [49].

Protective ω-3 fatty acids can also be released by PLA2G6. Dennis et al. has shown that the release of ω-3 and ω-6 fatty acids by PLA2G6 depends on their availability in the plasma membrane [50]. In fact, reducing the ω-6/ω-3 ratio has been shown to protect INS-1 β cells against cytokine-induced apoptosis [46]. Moreover, increased content of ω-3 fatty acids fatty acids in erythrocyte membranes has been shown to be associated with reduced risk of developing islet autoimmunity [47]. Therefore, shifting the fatty acid composition on the β-cell membrane may shift the balance towards protection against apoptotic signaling. Diet containing ω-3 fatty acids reduces the risk of islet autoimmunity in children [15] and the incidence of diabetes in NOD mice [14]. ω-3 fatty acids have been shown to reduce activation of T cells and macrophages [14, 51] and to ameliorate the intestinal barrier [52] in NOD mice, but their action on β cells is not well understood.

**Fig. 8:**
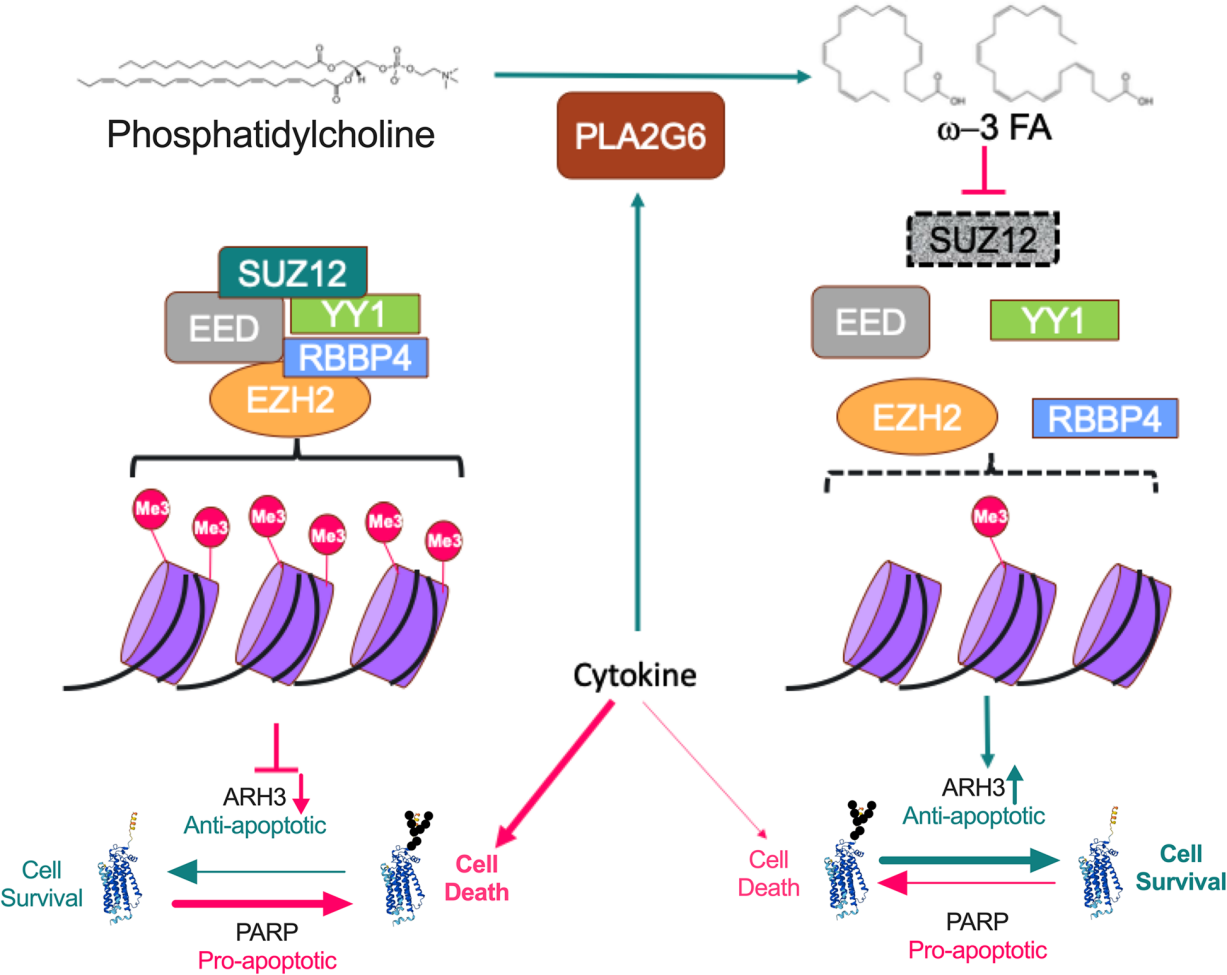
Protective mechanism of ω-3 fatty acids against cytokine-induced apoptosis. The schematic model represents cytokine-mediated hydrolysis of phosphatidylcholine by PLA2G6, giving rise to ω-3 fatty acids, which protect β cells by reducing ADP-ribosylation through upregulating ARH3 expression via SUZ12

Here we show that ω-3 fatty acids regulate antiapoptotic signals by downregulating SUZ12, a component of the histone methylation polycomb PCR2, and upregulating the expression of the ADP-ribosylhydrolase ARH3 (Fig. 8). This protection was at least partially dependent on PLA2G6, indicating that at least part of the ω-3 fatty acids are released from membranes. Regarding ARH3, it hydrolyzes ADP-ribosylation from serine residues, counterbalancing the pro-apoptotic activity induced by ADP-ribose polymerization during oxidative stress [53–55]. Indeed, ADP-ribosylation has a role in β-cell death; in murine islets, PARP1 induces cytokine-mediated β-cell death, and its deletion protects against streptozotocin-mediated diabetes [35, 56, 57]. Our data show that this scenario might be more complex as PARP3, − 9, − 10, − 12, and − 14 are strongly upregulated in cells treated with CT2. PARP-1-, PARP-9 and PARP14-mediated ADP-ribosylation enhance the activity of central pro-inflammatory transcription factors, such as NF-κB, NFATc3, and STAT1, in immune cells, playing an important role in the inflammatory signaling [58–60]. However, the role of the different proteins of the ADP-ribosylation machinery in β-cell death still needs to be further studied.

In summary, our data showed a consistent regulation of lipid metabolism in 3 different models of insulitis, including increase of PCs with PUFAs and LPCs, and decrease of TGs. These PCs with PUFAs were enriched in ω-3 fatty acids, which led us to study possible effects of their release by phospholipases, in this case PLA2G6. We demonstrated that phospholipase PLA2G6/ω-3 fatty acid signaling leads to the downregulation of SUZ12 and consequent upregulation of the ADP-ribosylhydrolase ARH3, which in turn reduces cytokine-induced apoptosis of MIN6 cells. These findings shed a light on the mechanism on how ω-3 fatty acids protect β cells and reduce the development of T1D.

### Supplementary Information

**Additional file 1: Figure S1.** Abundance of selected proteins from proteomics analysis of 3 common models used for the study of β-cell stress in type 1 diabetes: A EndoC-βH1 cells exposed to IL-1β and INF-γ for 48 h (*n =* 3), B human islets exposed to same cytokines for 24 h (*n =* 10) and C islets from non-obese diabetic (NOD) mice in pre-diabetic stage (6 weeks of age) vs. age-matched NOR mice (*n =* 3). Abbreviations: GBP2: interferon-induced guanylate-binding protein 2, Stat1: signal transducer and activator of transcription 1, TAP1: antigen peptide transporter 1. Statistical test: ** *p* ≤ 0.01 and *** *p ≤* 0.001 by *t*-test considering equal distribution and variance.

**Additional file 2: Table S1.** Characteristics of human islet donors. **Table S2.** Data collection parameters. **Table S3.** List of antibodies and oligonucleotides used in the study. **Table S4.** Quantitative proteomics analysis of islets from NOD and NOR mice at 6 weeks of age. **Table S5.** Differentially abundant proteins found in the quantitative proteomics analysis of islets from NOD and NOR mice at 6 weeks of age. **Table S6.** Quantitative lipidomics analysis in the negative-ionization mode of the EndoC-βH1 cells (*n =* 4) treated with 50 U/mL IL-1β + 1000 U/mL IFN-γ for 48 h. **Table S7.** Quantitative lipidomics analysis in the positive-ionization mode of the EndoC-βH1 cells (*n =* 4) treated with 50 U/mL IL-1β + 1000 U/mL IFN-γ for 48 h. **Table S8.** Quantitative lipidomics analysis in the negative-ionization mode of the human islets (*n =* 10) treated with 50 U/mL IL-1β + 1000 U/mL IFN-γ for 24 h. **Table S9.** Quantitative lipidomics analysis in the positive-ionization mode of the human islets (*n =* 10) treated with 50 U/mL IL-1β + 1000 U/mL IFN-γ for 24 h. **Table S10.** Quantitative lipidomics analysis in the negative-ionization mode of islets from 6-weeks old NOD vs. NOR mice. **Table S11.** Quantitative lipidomics analysis in the positive-ionization mode of islets from 6-weeks old NOD vs. NOR mice. **Table S12.** Quantitative proteomics analysis of wild-type and Pla2g6 knockdown MIN6 beta cells treated with cytokines (100 ng/mL IFN-γ, 10 ng/mL TNF-α, and 5 ng/mL IL-1β) for 24 h. **Table S13.** Identification of proteins that the abundances are regulated by cytokines via PLA2G6. Wild-type and Pla2g6 knockdown MIN6 beta cells treated with cytokine cocktail CT2 (100 ng/mL IFN-γ, 10 ng/mL TNF-α, and 5 ng/mL IL-1β) for 24 h were analyzed by proteomics. The criteria for selecting PLA2G6-dependent cytokine-regulated proteins were: (i) significantly regulated by the cytokine treatment (untreated wild type vs. wild type treated with cytokines), (ii) this regulation also had to be significant comparing wild type treated with cytokines vs. Pla2g6 knockdown treated with cytokines, and (iii) not significantly changing in untreated Pla2g6 knockdown vs. Pla2g6 knockdown treated with cytokines.

## Data Availability

Proteomics data were deposited into the Pride repository (www.ebi.ac.uk/pride) under accession number PXD017863, PXD021501 and PXD021475. Lipidomics data were deposited into the Massive repository (http://massive.ucsd.edu/) under accession number MSV000086174. Pla2g6-dependent cytokine signaling in MIN6 cells. Project number: PXD017863. Reviewer account details: Username: reviewer04836@ebi.ac.uk Password: B4o1VcPd. ARH3-regulated cytokine signaling in MIN6 cells. Project accession: PXD021501. Reviewer account details: Username: reviewer_pxd021501@ebi.ac.uk Password: NbhKnd9N. Proteomics analysis of islets of 6-week old NOR and NOD mice. Project accession: PXD021475. Reviewer account details: Username: reviewer_pxd021475@ebi.ac.uk Password: tKFfLzrh. Lipidomics of 3 models of insulitis, beta-cell stress and type 1 diabetes development. Project accession: MSV000086174. Reviewer account details: Username: MSV000086174_reviewer. Password: Islets3663.

## Abbreviations

FISH: Fluorescent in situ hybridization
GPC: Glycerophosphocholine
KD: Knockdown
LPC: Lysophosphatidylcholine
MPLEx: Metabolite, protein, and lipid extraction
MSI: Mass spectrometry imaging
NOD: Non-obese diabetic (mouse)
NOR: Non-obese diabetes-resistant (mouse)
PC: Phosphatidylcholine
PCA: Principal component analysis
PG: Phosphatidylglycerol
PLA2: Phospholipase A2
OCT: Optimal cutting temperature
T1D: Type 1 diabetes
TG: Triacylglycerol
