# Supplementary material for: Regulation of β-cell death by ADP-ribosylhydrolase ARH3 via lipid signaling in insulitis
Source: Cell Commun Signal. 2024 Feb 21;22:141. doi: 10.1186/s12964-023-01437-1 (PMC10880366; doi:10.1186/s12964-023-01437-1)
Supplement: Supplementary file 2 — Additional file 1: Figure S1. Abundance of selected proteins from proteomics analysis of 3 common models used for the study of β-cell stress in type 1 diabetes: A EndoC-βH1 cells exposed to IL-1β and INF-γ for 48 h (n = 3), B human islets exposed to same cytokines for 24 h (n = 10) and C islets from non-obese diabetic (NOD) mice in pre-diabetic stage (6 weeks of age) vs. age-matched NOR mice (n = 3). Abbreviations: GBP2: interferon-induced guanylate-binding protein 2, Stat1: signal transducer and activator of transcription 1, TAP1: antigen peptide transporter 1. Statistical test: ** p ≤ 0.01 and *** p ≤ 0.001 by t-test considering equal distribution and variance. [file 12964_2023_1437_MOESM1_ESM.docx]

**Supplemental figure**

**Regulation of β-cell death by ADP-ribosylhydrolase ARH3 via lipid signaling in insulitis**

Soumyadeep Sarkar^1^, Cailin Deiter^2^, Jennifer E. Kyle^1^, Michelle A. Guney^2^, Dylan Sarbaugh^2^, Ruichuan Yin^3^, Xiangtang Li^3^, Yi Cui^5^, Mireia Ramos-Rodriguez^10^, Carrie D. Nicora^1^, Farooq Syed^6^, Jonas Juan-Mateu^7,8^, Charanya Muralidharan^9^, Lorenzo Pasquali^10^, Carmella Evans-Molina^6^, Decio L. Eizirik^7^, Bobbie-Jo M. Webb-Robertson^1,11^, Kristin Burnum-Johnson^4^, Galya Orr^4^, Julia Laskin^3^, Thomas O. Metz^1^, Raghavendra G. Mirmira^9^, Lori Sussel^2^, Charles Ansong^1^, Ernesto S. Nakayasu^1,*^

^1^Biological Sciences Division, Pacific Northwest National Laboratory, Richland, WA, 99354, USA

^2^Barbara Davis Center for Diabetes, University of Colorado Anschutz Medical Center, Aurora, CO, 80045, USA

^3^Department of Chemistry, Purdue University, West Lafayette, IN, 47907-2084, USA

^4^Environmental and Molecular Sciences Laboratory, Pacific Northwest National Laboratory, Richland, WA, 99354, USA

^5^NanoString Technologies, Seattle, WA, 98109, USA

^6^Center for Diabetes and Metabolic Diseases and the Herman B Wells Center for Pediatric Research, Indiana University School of Medicine, Indianapolis, IN, 46202, USA

^7^ULB Center for Diabetes Research, Université Libre de Bruxelles (ULB), 1070, Brussels, Belgium

^8^Centre for Genomic Regulation (CRG), The Barcelona Institute of Science and Technology, 08003, Barcelona, Spain

^9^Kovler Diabetes Center and Department of Medicine, The University of Chicago, Chicago, IL, 60637, USA

^10^Endocrine Regulatory Genomics, Department of Experimental & Health Sciences, University Pompeu Fabra, 08003, Barcelona, Spain

^11^Department of Biostatistics and Informatics, University of Colorado Anschutz Medical Center, Aurora, CO, 80045, USA

**Figure S1**


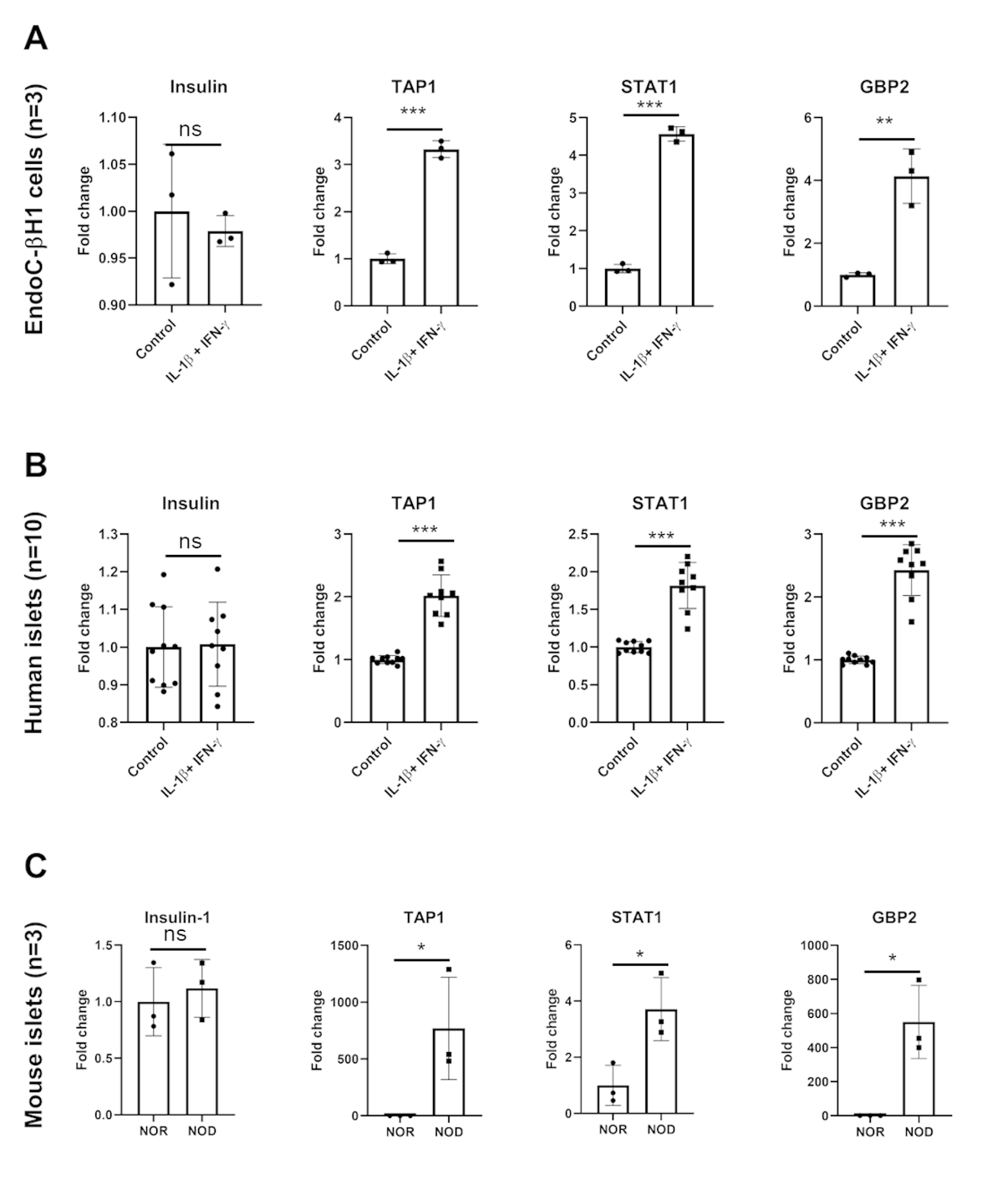


**Figure S1**. Abundance of selected proteins from proteomics analysis of 3 common models used for the study of β-cell stress in type 1 diabetes: **A** EndoC-βH1 cells exposed to IL-1β and INF-γ for 48 h (n=3), **B** human islets exposed to same cytokines for 24 h (n=10) and **C** islets from non-obese diabetic (NOD) mice in pre-diabetic stage (6 weeks of age) vs. age-matched NOR mice (n=3). Abbreviations: GBP2: interferon-induced guanylate-binding protein 2, Stat1: signal transducer and activator of transcription 1, TAP1: antigen peptide transporter 1. Statistical test: ** p ≤0.01 and *** p≤0.001 by *t*-test considering equal distribution and variance.
